# Key role of the CCR2-CCL2 axis in disease modification in a mouse model of tauopathy

**DOI:** 10.1186/s13024-021-00458-z

**Published:** 2021-06-25

**Authors:** Hila Ben-Yehuda, Michal Arad, Javier María Peralta Ramos, Efrat Sharon, Giulia Castellani, Shir Ferrera, Liora Cahalon, Sarah Phoebeluc Colaiuta, Tomer-Meir Salame, Michal Schwartz

**Affiliations:** 1 Department of Neurobiology, Weizmann Institute of Science, Rehovot, Israel; 2 Flow Cytometry Unit, Life Science Core Facilities, Weizmann Institute of Science, Rehovot, Israel

**Keywords:** Tauopathy, Immunotherapy, CCR2, CCL2, Monocytes, Regulatory T cells, PD-L1, Dementia, CXCL12

## Abstract

**Background:**

For decades, dementia has been characterized by accumulation of waste in the brain and low-grade inflammation. Over the years, emerging studies highlighted the involvement of the immune system in neurodegenerative disease emergence and severity. Numerous studies in animal models of amyloidosis demonstrated the beneficial role of monocyte-derived macrophages in mitigating the disease, though less is known regarding tauopathy. Boosting the immune system in animal models of both amyloidosis and tauopathy, resulted in improved cognitive performance and in a reduction of pathological manifestations. However, a full understanding of the chain of events that is involved, starting from the activation of the immune system, and leading to disease mitigation, remained elusive. Here, we hypothesized that the brain-immune communication pathway that is needed to be activated to combat tauopathy involves monocyte mobilization via the C-C chemokine receptor 2 (CCR2)/CCL2 axis, and additional immune cells, such as CD4^+^ T cells, including FOXP3^+^ regulatory CD4^+^ T cells.

**Methods:**

We used DM-hTAU transgenic mice, a mouse model of tauopathy, and applied an approach that boosts the immune system, via blocking the inhibitory Programmed cell death protein-1 (PD-1)/PD-L1 pathway, a manipulation previously shown to alleviate disease symptoms and pathology. An anti-CCR2 monoclonal antibody (αCCR2), was used to block the CCR2 axis in a protocol that partially eliminates monocytes from the circulation at the time of anti-PD-L1 antibody (αPD-L1) injection, and for the critical period of their recruitment into the brain following treatment.

**Results:**

Performance of DM-hTAU mice in short-term and working memory tasks, revealed that the beneficial effect of αPD-L1, assessed 1 month after a single injection, was abrogated following blockade of CCR2. This was accompanied by the loss of the beneficial effect on disease pathology, assessed by measurement of cortical aggregated human tau load using Homogeneous Time Resolved Fluorescence-based immunoassay, and by evaluation of hippocampal neuronal survival. Using both multiparametric flow cytometry, and Cytometry by Time Of Flight, we further demonstrated the accumulation of FOXP3^+^ regulatory CD4^+^ T cells in the brain, 12 days following the treatment, which was absent subsequent to CCR2 blockade. In addition, measurement of hippocampal levels of the T-cell chemoattractant, C-X-C motif chemokine ligand 12 (*Cxcl12)*, and of inflammatory cytokines, revealed that αPD-L1 treatment reduced their expression, while blocking CCR2 reversed this effect.

**Conclusions:**

The CCR2/CCL2 axis is required to modify pathology using PD-L1 blockade in a mouse model of tauopathy. This modification involves, in addition to monocytes, the accumulation of FOXP3^+^ regulatory CD4^+^ T cells in the brain, and the T-cell chemoattractant, *Cxcl12*.

**Supplementary Information:**

The online version contains supplementary material available at 10.1186/s13024-021-00458-z.

## Background

Dementia is describing conditions associated with cognitive loss, affecting millions of people worldwide. Some of the most common types of dementias include Alzheimer’s disease (AD), dementia with Lewy bodies, frontotemporal dementia, and Parkinson’s disease [1–3]. Numerous studies have shown that the fate of the central nervous system (CNS) in homeostasis and pathology is influenced by circulating immune cells [4]. In homeostasis, it was shown that systemic immune cells are needed for healthy brain plasticity [5–7]. In acute pathology, such as CNS injury, monocyte-derived macrophages, CD4^+^ T cells, and FOXP3^+^ regulatory CD4^+^ T cells (Tregs) were found to be recruited into the damaged tissue and to promote its repair [8–10]. Based on these findings, our group [11] and others [12–18] sought an approach to induce the recruitment of peripheral leukocytes into the brains of mouse models of AD and dementia. Harnessing the peripheral immune response, by transiently blocking the inhibitory immune checkpoint pathway Programmed cell death protein-1 (PD-1)/PD-L1, resulted in an improved cognitive performance and in a reduction of pathological manifestations [11, 19]. Yet, a full understanding of the chain of events that must be activated to combat the disease remained unresolved.

Here, we hypothesized that the C-C chemokine receptor 2 (CCR2)/CCL2 axis might be involved in the therapeutic effect in tauopathy, by facilitating recruitment of CCR2^+^ monocytes. We further hypothesized that these monocytes could serve as phagocytic cells and producers of cytokines [14, 19–23], and would also orchestrate accumulation of additional immune-regulating cells, such as Tregs [8], which were shown to be involved in protecting the CNS under neurodegenerative conditions [24, 25].

To address the role of the CCR2 axis, we administered PD-L1 immunotherapy in a mouse model of tauopathy, and found that a single systemic injection of anti-PD-L1 antibody (αPD-L1) led to upregulation of CCR2^+^ monocytes and Tregs in the peripheral blood. To block the CCR2 axis, we used an antibody specific to C-C chemokine receptor type 2 (CCR2), known to play a critical role in monocyte egress from the bone marrow to the blood, and in their homing into damaged tissues, including the brain [19–22]. Blocking CCR2 abrogated the beneficial effect of the treatment on cognitive behavior, disease pathology, neuronal survival, and hippocampal inflammation. In addition, CCR2 blockade prevented the accumulation of Tregs in the brain, which we observed here after αPD-L1 treatment. In addition, we found changes in hippocampal expression of C-X-C motif chemokine ligand 12 (*Cxcl12)*, a strong chemoattractant of lymphocytes and specifically of Tregs [26, 27]; reduction in *Cxcl12* levels correlated with the effect of αPD-L1 on inflammation. Altogether, our results suggest that the CCR2/CCL2 axis is an important player in brain-immune communication, needed for modifying tauopathy.

## Methods

### Animals

Heterozygous DM-hTAU transgenic mice, bearing two mutations (K257T/P301S) in the human-tau (hTAU) gene (double mutant, DM; on a BALBc-C57/BL6 background), associated with severe disease manifestations of frontotemporal-dementia in humans [28], expressed under the natural tau promoter, were used throughout the study. Non-transgenic littermates from the same colony were used as wild-type (WT) controls. Both sexes were used (unless otherwise is mentioned in figure legends), and were equally distributed between the experimental groups. Throughout the study, 8–13 month old mice were used. For cognitive assessments, the mice were kept in a reverse light-dark cycle (lights off: 9:00–21:00) with ad-libitum food and water. Mice were bred and maintained at the Animal Breeding Center of the Weizmann Institute of Science. All experiments complied with the regulations formulated by the Institutional Animal Care and Use Committee of the Weizmann Institute of Science.

### Treatment with antibodies

Anti-PD-L1 (1.5 mg; rat IgG2b isotype; clone 10F.9G2; BioXCell; BE0101) or isotype control (1.5 mg; anti-keyhole limpet hemocyanin; clone LTF-2; BioXCell; BE0090) antibodies were administered intraperitoneally (i.p.).

For CCR2 blockade, the anti-CCR2 monoclonal antibody MC21 [29], was injected i.p. (400 μg) every 4 days.

### Flow cytometry

Blood: Blood was collected from the mice, and red blood cells were lysed using ACK Lysis Buffer (Gibco; A1049201). The samples were then washed with PBS, incubated with Fc-block CD16/32 (BioLegend Inc.; 101302), and subsequently stained.

Spleen: The mice were perfused with PBS, and spleens were excised. The excised tissues were mechanicaly dissociated to form single-cell suspentions. Red blood cells were lysed using ACK Lysis Buffer (Gibco; A1049201). The samples were then washed with PBS, incubated with Fc-block CD16/32 (BioLegend Inc.; 101302), and subsequently stained.

Brain: Following perfusion with PBS, and brains were excised. Single-cell suspensions were obtained using a software-controlled sealed homogenization system (Dispomix®, Medic Tools; Miltenyi) in PBS. For density gradient separation, the single-cell suspension was mixed with 40% Percoll (Cytiva; 17089101) and centrifuged at 800 g for 20 min at room temperature. The samples were then washed with PBS, incubated with Fc-block CD16/32 (BioLegend Inc.; 101302), and subsequently stained. To detect Tregs in the brain, 10 brains per group were pooled together after density gradient isolation. These experiments were repeated twice and results were combined.

For FOXP3 staining, the samples were fixed, permeabilized, and subsequently stained using FOXP3/Transcription Factor Staining Buffer Set (eBioscience; 00–5523-00), according to the manufacturer’s instructions.

The following antibodies were used: APC- conjugated CD44 (103012), APC- conjugated Ly6G (127614), APC/Cy7- conjugated Ly6G (127624), APC/Cy7- conjugated TCRβ (109220), Alexa 700- conjugated CD44 (103026), BV421- conjugated CD45 (103133), BV421- conjugated CD4 (100544), BV650- conjugated MHCII (107641), FITC- conjugated CD11b (101206), FITC- conjugated CD45 (103108), FITC- conjugated CCR2 (150608), PE- conjugated CD3ε (100308), PE- conjugated CD4 (100408), PE- conjugated CCR2 (150610), PE/Cy7- conjugated CD45 (103114), PE/Dazzle 594- conjugated CD38 (102730), PE/Dazzle 594- conjugated CD44 (103056), PerCP/Cy5.5- conjugated CD62L (104432) [all from BioLegend Inc.]; APC- conjugated FOXP3 (17–5773-82), PE- conjugated CD115 (135506), and PerCP/Cy5.5- conjugated Ly6C (45–5932-82) [all from eBioscience, Inc.].

The samples were analyzed on a FACS-LSRII cytometer (BD Biosciences) using BD FACSDIVA software (BD Biosciences), or on a CytoFLEX-S Flow Cytometer (Beckman Coulter) using CytExpert software (Beckman Coulter), and data were processed by FlowJo (FlowJo, LLC) software.

### Cognitive assessment

To assess cognitive performance, mice were taken through a battery of three consecutive tasks (described below), assessing a variety of cognitive capabilities. Each mouse was subjected to a daily 3 min handling session for 5 successive days prior to the first behavioral test.

Behavioral studies were repeated twice and results were combined. The investigators performing behavioral testing were blinded to the treatment group of the mice throughout the experiments. Testing sessions were recorded and analyzed using EthoVision tracking system XT 11 (Noldus Information Technology), and the identity of the mice was un-coded for statistical analyses by a member of the team who did not perform the behavioral tests.

### T-maze

The T-maze test assesses spatial short-term memory and alternation behavior, and is based on the natural willingness of the mice to explore unfamiliar territories. Therefore, this procedure allows the evaluation of the animal’s ability to recognize and differentiate between a novel unknown versus a familiar compartment [30]. The T-shaped maze was made of plastic with two 45 cm long arms, which extended at right-angles from a 57 cm long alley. The arms had a width of 10 cm and were surrounded by 10 cm high walls. The task consisted of two trials with a 5 min inter-trial interval (ITI), during which mice were kept in a single-housed holding cage. Mice were returned to their home-cages at the end of the test. Acquisition trial – 8 min: Each mouse was lowered to the center of the maze, facing the wall, and allowed to freely explore two of the maze’s arms, while the third arm was inaccessible. Retention trial – 3 min: With all three arms accessible, each mouse was allowed to freely explore the entire maze. Cognitively healthy mice tend to prefer the novel arm over the familiar arms. This was calculated as: percent novel arm exploration = 100 × (time spent in novel arm)/(time spent in novel arm + time spent in familiar arms).

### Y-maze

Spontaneous alternation behavior was recorded in a Y-maze to assess short-term memory performance [31]. The apparatus was a symmetrical Y-maze; each arm measured 50 × 10 cm, with 40 cm high side walls. Mice were placed in the maze, and allowed to freely explore for 5 min. Arms were arbitrarily labeled A, B, and C, and the sequence of arm entries was used to determine alternation behavior. An alternation was defined as a triad consisting of consecutive entries into each of the three arms. The number of maximum alternations was therefore the total number of arm entries minus two, and the percentage of alternations was calculated as (actual alternations/maximum alternations) × 100. For example, for arms referred to as A, B, C, if the mouse performed ABCABCABBAB, the number of arm entries would be 11, and the successive alternations: ABC, BCA, CAB, ABC, BCA, CAB. Therefore, the percent spontaneous alternation = [6/(11–2)] × 100 = 66.7% [32].

### Novel object recognition (NOR)

The novel object recognition test provides an index of recognition memory [33]. Modified from Bevins and Besheer, 2016 [34], a square gray box (45 × 45 × 50 cm) with visual cues on the walls was used. The task spanned 2 days and three trials: a habituation trial – a 20 min session in the empty apparatus (day 1), a familiarization trial – a 10 min session allowing the mice to interact with two identical objects (day 2), and a test trial: following a 1 h ITI, each mouse was returned to the apparatus for a 6 min session in which one of the objects was replaced by a novel one. Novel object preference was calculated as: percent novel object exploration = ((novel object exploration time)/(novel object exploration time + familiar object exploration time)) × 100.

### Assessment of hyperphosphorylated human tau by ELISA

After perfusion, mouse hippocampus was dissected and homogenized in 5 M guanidine-HCl diluted in 50 mM Tris, pH 8.0, followed by incubation on an orbital shaker at room temperature for 4 h. The samples then were diluted ten-fold with cold PBS supplemented with protease inhibitor cocktail (Sigma; P8340) and phosphatase inhibitor cocktail (PhosSTOP, Roche; 04906845001), centrifuged (16,000 g, 20 min, 4°c) and the supernatant was aliquoted and frozen (− 80 °C). For measuring human tau pS199, a commercially available ELISA kit (Invitrogen; KHB7041) was used, according to the manufacturer’s instructions, with two modifications: (1) 80 μl from each sample was loaded and combined with 20 μl of the Standard Diluent Buffer. (2) The serial dilution of the Hu Tau [pS199] Standard was continued to a concentration of 7.8 pg/ml (Std8). Protein concentrations were measured using BCA protein assay kit (Pierce; 23227), according to the manufacturer’s instructions. Human tau pS199 levels were calculated based on a four parameter algorithm, and were normalized to the total protein concentration.

### Aggregated human tau measurement by HTRF

After perfusion, mouse cortices were dissected and homogenized in ice-cold buffer (349.1 mM sucrose, 0.1 mM CaCl_2_, 1 mM MgCl_2_) supplemented with a protease inhibitor cocktail (Sigma; P8340), incubated on ice (10 min), and centrifuged (16,000 g, 10 min, 4 °C). The supernatant was collected, diluted in TBS with 1% Triton X-100 (Sigma; X100) supplemented with a protease inhibitor cocktail, and was tested for the presence of aggregated human Tau by Homogeneous Time Resolved Fluorescence (HTRF), using a commercially available kit (CisBio; 6FTAUPEG), according to the manufacturer’s instructions. This assay is based on a fluorescence resonance energy transfer (FRET) measurement. Protein concentrations were measured using BCA protein assay kit (Pierce; 23227), according to the manufacturer’s instructions. Aggregated human tau levels were calculated as Delta F%, and were normalized to the protein concentration. The experiments were performed with two technical repeats.

### Cresyl violet staining

Following perfusion, brains were excised and fixed. Tissue processing and Cresyl violet staining were performed on paraffin-embedded sections (6 μm thick). Cresyl Echt Violet Solution (0.1%) [ScyTek; CEA999] was used. Pyramidal neurons were counted in each brain from five serial sections located 90 μm apart. All cell counting was performed by a researcher who was blind to the identity of the animals.

### Cytometry by time of flight (CyTOF)

Following anesthesia, blood was collected via cardiac puncture, mice were perfused, and brains were harvested. Single-cell suspension of blood samples was obtained by isolation of the mononuclear cells by Ficoll-Paque [(GE Healthcare; 17544602) 400 g, 30 min, 20°c]. Red blood cells were lysed using ACK Lysis Buffer (Gibco; A1049201). Single-cell suspension of brain samples was obtained by mechanical dissociation, followed by incubation with an enzyme cocktail [RPMI supplemented with 0.4 mg/ml Collagenase Type 4 (Worthington Biochemical Corporation; LS004188), 2 mM HEPES (BI Industries; 03–025-1B), 10 μg/ml DNase I (Roche; 10104159001), and 2% FCS] for 30 min at 37 °C, and homogenization using syringes with 21G needles. Enzymatic activity was stopped with cold RPMI supplemented with 0.5 M EDTA (0.2%), and the cells were isolated by 30% Percoll (Cytiva; 17089101) density gradient centrifugation (23,500 g, 30 min, 4 °C). Both blood and brain samples were stained by 1.25 μM Cell-ID Cisplatin (Fluidigm; 201064) in MaxPar Cell Staining Buffer (Fluidigm; 201068) and washed twice with warmed RPMI supplemented with 10% FBS, followed by MaxPar Cell Staining Buffer. The samples were then incubated with Fc-block CD16/32 [(BioLegend Inc.; 101302) 10 min, RT], followed by the addition of the extracellular antibodies (30 min, RT). Brain samples were fixed and barcoded using the Cell-ID 20-Plex Pd Barcoding Kit (Fluidigm; 201060), according to the manufacturer’s instructions, and then combined into a composite sample, resuspended with 4% Formaldehyde (Pierce; 28906), and kept at 4 °C.

Blood samples were fixed and permeabilized using the FOXP3/Transcription Factor Staining Buffer Set (eBioscience; 00–5523-00), barcoded using the Cell-ID 20-Plex Pd Barcoding Kit (Fluidigm; 201060), and combined into a composite sample. Intracellular staining was performed in the presence of Permeabilization buffer (eBioscience; 00–8333-56), followed by fixation with 4% Formaldehyde (Pierce; 28906).

On the day of reading, samples were labeled with Cell-ID Intercalator-Ir (Fluidigm; 201192A) according to the manufacturer’s instructions, washed with MaxPar Cell Staining Buffer and MaxPar water (Fluidigm; 201069), followed by acquisition using a CyTOF 2 analyzer upgraded to Helios system (Fluidigm). A detailed list of antibodies used is supplied in the Supplementary section (Additional file 1). Purified antibodies were conjugated using Maxpar X8 Antibody Labeling Kit (Fluidigm) or by Lightning Link Metal Labeling Kit (Expedeon), as detailed in the Supplementary section (Additional file 1).

### CyTOF data analysis

CyTOF data were analyzed using the R Cytofkit package [35]. The data were transformed using the “arcsinh” (inverse hyperbolic sine) parameter, and up to 6300 CD45^+^ cells per sample were further processed. Two-dimensional reduction was applied using the Cytofkit “tsne” (T-distributed Stochastic Neighbor Embedding; t-SNE) method, and FlowSOM-based clustering was performed. To combine the data from the three different experimental repetitions, the percentage of cells in each cluster per mouse was calculated, and is presented as a ratio relative to the average of the control IgG group.

### RT-qPCR

Following perfusion, hippocampus was dissected. Total RNA was extracted with the RNeasy Mini kit according to the manufacturer’s instructions (Qiagen; 74104). RNA was reverse-transcribed using the High-capacity cDNA reverse transcription kit (Applied Biosystems; 4368814), amplified using Fast SYBR green Master Mix (Applied Biosystems; 4385614) and detected by StepOnePlus (Applied Biosystems), in duplicates. Results were normalized to the expression of the housekeeping gene, *Peptidylprolyl isomerase-a* (*Ppia*), and then expressed as a ratio relative to the control samples. The following primers were used:

- *Ppia* forward, 5′-AGCATACAGGTCCTGGCATCTTGT-3*'*, and reverse, 5′-CAAAGACCACATGCTTGCCATCCA-3′;
- *Il-1β* forward, 5′-ACCTGTCCTGTGTAATGAAAGAC-3′ and reverse, 5′-TGGGTATTGCTTGGGATCCA-3′.
- *Tnfα* forward, 5′-CCCTCACACTCAGATCATCTTCT-3′, and reverse, 5′-GCTACGACGTGGGCTACAG-3′;
- *Il-6* forward, 5′-TGCAAGAGACTTCCATCCAGTTG-3′ and reverse, 5′-TAAGCCTCCGACTTGTCAAGTGGT-3′.
- *Il-12p35* forward, 5′-TCACCCTGTTGATGGTCACG-3′ and reverse, 5′-AAATGAAGCTCTGCATCCTGC-3′.
- *Cxcl12* forward, 5′-CATCAGTGACGGTAAACCAG-3′ and reverse, 5′-TTTCAGATGCTTGACGTTGG-3′.

### Statistics

Two-tailed Student’s *t*-tests or one-way ANOVA with *post-hoc* tests were used, as indicated in the figure legends. Results are presented as mean ± s.e.m. *p < 0.05, **p < 0.01, ***p < 0.001. Statistical calculations were performed using GraphPad Prism software (GraphPad Software, San Diego, CA). Sample sizes were chosen with adequate statistical power based on the literature and our previous experience. Data points were excluded only if they differed by at least 2 standard deviations from the average.

## Results

### Anti-CCR2 antibody reduces the frequency of peripheral myeloid cell populations without affecting cognitive behavior

**Fig. 1:**
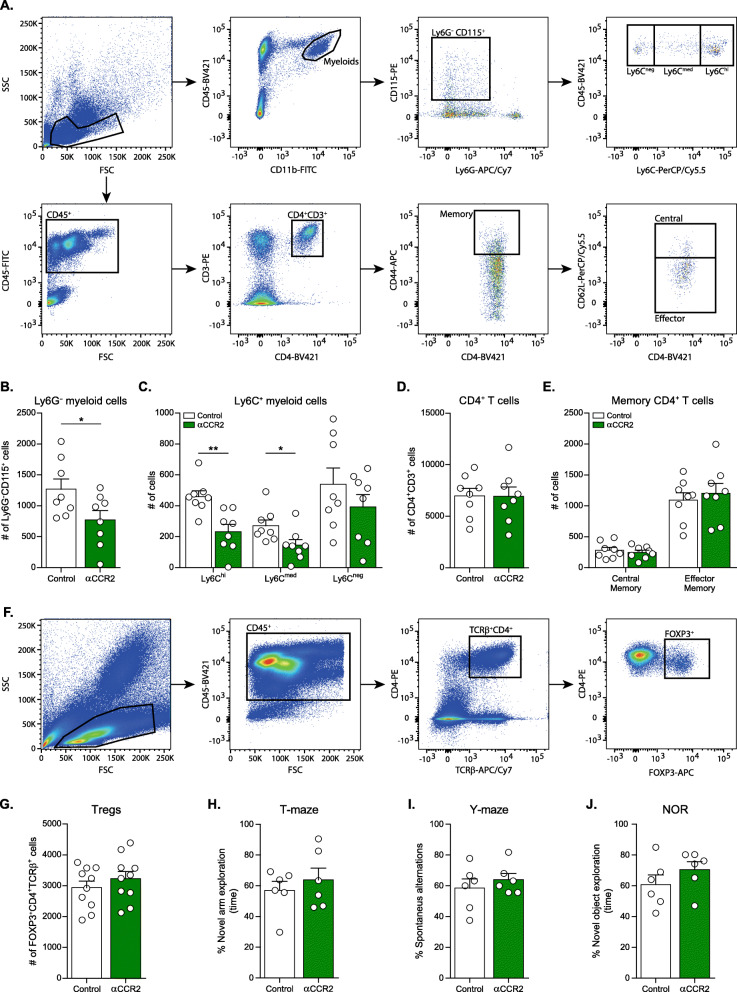
Anti-CCR2 antibody reduces monocyte populations in the blood without affecting cognitive behavior. WT mice received 4 injections of αCCR2 antibody every 4 days, while control mice were untreated. Mice were euthanized 3 days after the 4th injection, and blood was collected. (**A**) Flow cytometry gating strategy for monocyte (top) and memory CD4^+^ T cell (bottom) populations in the blood of WT animals. (**B**) Flow cytometry analyses of Ly6G^−^ CD115^+^ myeloid cells (two-tailed Student’s *t*-test: t_(14)_ = 2.256, *p = 0.0406), (**C**) Ly6C^+^ myeloid populations (two-tailed Student’s *t*-test: Ly6C^hi^ t_(14)_ = 3.764, **p = 0.0021*;* Ly6C^med^ t_(14)_ = 2.442, *p = 0.0285), (**D**) CD4^+^ T cells, and (**E**) memory CD4^+^ T cell populations in control and αCCR2-injected groups, *n* = 8 mice per group. (**F**) Flow cytometry gating strategy and (**G**) analysis of Tregs in the blood of WT mice, *n* = 10 mice per group. Data are presented as mean ± s.e.m. *p < 0.05, **p < 0.01. (**H-J**) Male WT mice received 4–5 injections of αCCR2 antibody every 4 days, while control mice remained untreated. Behavioral testing was carried out during the 4 days following the last injection. (**H**) T-maze: Willingness to explore a novel environment is presented as percent of time spent by each mouse in a novel arm divided by the total time spent in all three arms (the novel arm and two familiar arms). (**I**) Spontaneous alternation in the Y maze: The spontaneous alternation behavior of the mice is presented as percent alternation: number of alternations divided by number of possible triads (see Methods). (**J**) Novel Object Recognition (NOR): The memory recognition is presented as percent time the mouse interacted with the novel object divided by the total time spent with both objects. *n* = 6 mice per group. Data are presented as mean ± s.e.m. One-way ANOVA was used for the analyses

To test the potential role of the CCR2/CCL2 axis in disease modification, we first established a methodology to selectively block CCR2^+^ immune cells, which are mostly comprised of activated monocytes [29, 36–38]. To this end, we used an anti-CCR2 antibody (αCCR2), which was previously demonstrated to efficiently deplete monocytes from the peripheral blood [29], and was found to be an effective tool to prevent the homing of monocytes into the CNS under various pathologies [39–42], and following immune activation [10]. Yet, since CCR2 can be expressed by other immune-cell types, including Tregs [43, 44] and effector memory CD4^+^ T cells [45], we tested the specificity of αCCR2. To this end, wild type (WT) mice were intraperitoneally (i.p.) injected with 400 μg of αCCR2 every 4 days, for a total of four injections, and 3 days after the last injection, blood was collected and analyzed by multiparametric flow cytometry (Fig. 1). We found that the number of Ly6G^−^ CD115^+^ myeloid cells was significantly reduced compared to their number in untreated control mice (Fig. 1A, B). Moreover, analysis of Ly6C-expressing cells revealed significantly reduced numbers of Ly6C^hi^ and Ly6C^med^ monocytes, relative to controls (Fig. 1C). In contrast, analyses of total CD4^+^ T cells and of memory- and regulatory- CD4^+^ T cell populations did not show any changes following αCCR2 administration (Fig. 1D-G). These results verified that αCCR2 effectively targets circulating monocytes, thereby reducing their numbers in the peripheral blood, without modifying CD4^+^ T cell populations.

To rule out the possibility that interfering with the CCR2/CCL2 axis would affect cognition, we next examined brain function in healthy mice. To this end, WT mice were administered αCCR2 every 4 days for a total of 4–5 injections, and during 4 days after the last injection the animals were tested using a battery of cognitive assessments including novel arm exploration test in a T-maze, examining short-term spatial memory and alternation behavior [30]; spontaneous alternation task in a Y-maze evaluating short-term memory performance [31]; and novel object recognition (NOR) paradigm which provides an index of recognition memory [33]. The mice showed an intact performance in all three cognitive paradigms tested, with no difference between αCCR2-injected mice and their control-untreated littermates (Fig. 1H-J). These results ruled out any possible non-specific negative effects of αCCR2 administration on cognitive activity in healthy animals. Together, these findings prompted us to further use this αCCR2 as a tool for deciphering the role of this axis in the repair process induced by PD-L1 immune checkpoint blockade.

### Anti-CCR2 antibody abrogates the beneficial effect of anti-PD-L1 antibody treatment in DM-hTAU mice

**Fig. 2:**
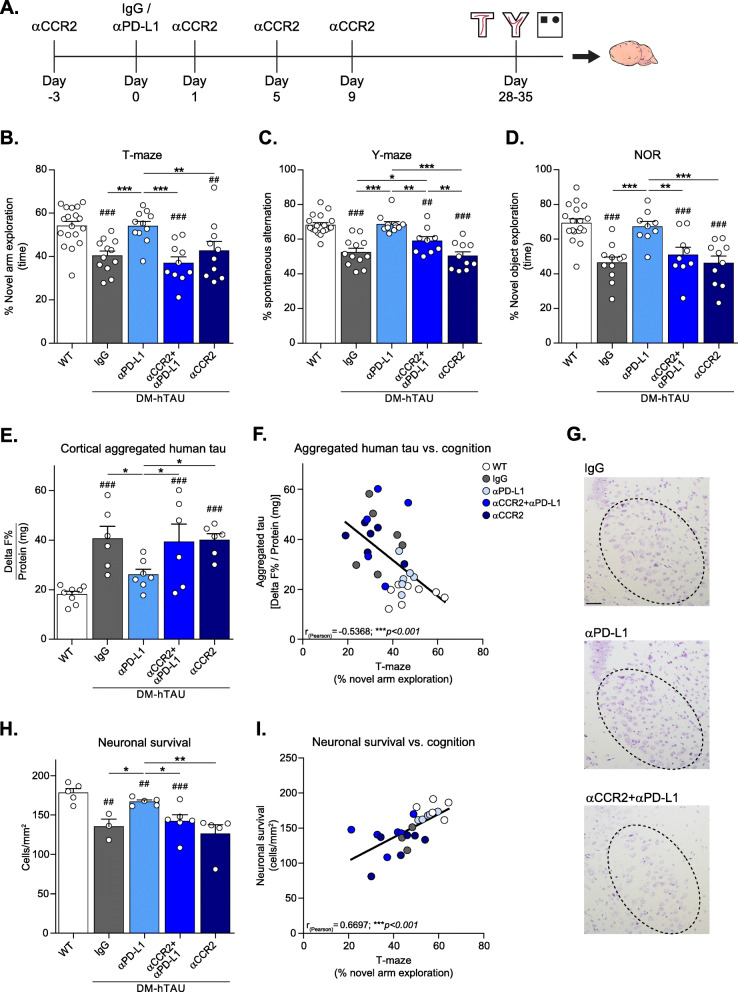
Anti-CCR2 antibody abrogates the beneficial effect of PD-L1 blockade. This experiment included four groups of DM-hTAU mice treated either with: IgG, αPD-L1, αCCR2 + αPD-L1, or αCCR2. An additional group of WT mice served as healthy controls. (**A**) Schematic presentation of experimental design: αCCR2 was i.p. injected to DM-hTAU mice 3 days prior (Day − 3) to αPD-L1 or IgG (Day 0), and again on days 1, 5 and 9. The cognitive behavior of the animals was assessed 1 month after αPD-L1 treatment by T-maze, spontaneous alternation test in Y-maze, and novel object recognition. Subsequently, brains were removed, and aggregated human tau protein levels in the cortices was measured. (**B**) T-maze: Willingness to explore a novel environment is presented as percent of time spent by each mouse in a novel arm divided by the total time spent in all three arms (novel and two familiar arms. One-way ANOVA F_(4,56)_ = 9.068, ***p < 0.0001). (**C**) Spontaneous alternation in Y maze: The spontaneous alternation behavior of the mice is presented as percent alternation: number of alternations divided by number of possible triads (see Methods. One-way ANOVA F_(4,55)_ = 19.73, ***p < 0.0001). (**D**) Novel Object Recognition (NOR): novel object preference is presented as the percent time the mouse interacted with the novel object divided by the total time spent with both objects (One-way ANOVA F_(4,52)_ = 12.48, ***p < 0.0001). (**B-D**). *Post-hoc* uncorrected Fisher’s LSD multiple comparisons between DM-hTAU groups to WT: #*p* < 0.05, ##*p* < 0.01, ###*p* < 0.001. *Post-hoc* uncorrected Fisher’s LSD multiple comparisons between the DM-hTAU groups: *p < 0.05, **p < 0.01, ***p < 0.001. *n* = 9–18 mice per group. Results were combined from two independent experiments. Data are presented as mean ± s.e.m. (**E**) Cortical aggregated human tau protein levels were measured by HTRF immunoassay, and are presented as Delta F% normalized to the amount of total protein in each tissue (mg). One-way ANOVA F_(4,28)_ = 7.409, ***p = 0.0003. *Post-hoc* uncorrected Fisher’s LSD multiple comparisons between DM-hTAU groups to WT: ###*p* < 0.001. *Post-hoc* uncorrected Fisher’s LSD multiple comparisons between the DM-hTAU groups: *p < 0.05. n = 8–6 mice per group. Data are presented as mean ± s.e.m. (**F**) Pearson correlation coefficient test between the measured aggregated human tau protein and the T maze score of each mouse revealed a significant negative linear correlation. r_(Pearson)_ = − 0.5368, ***p < 0.001. (**G**) Representative images (scale bar - 100 μm) and (**H**) quantification of pyramidal neurons in the subiculum of female mice. One-way ANOVA F_(4,19)_ = 7.686, ***p = 0.0007. *Post-hoc* uncorrected Fisher’s LSD multiple comparisons between DM-hTAU groups to WT: ##*p* < 0.01, ###*p* < 0.001. *Post-hoc* uncorrected Fisher’s LSD multiple comparisons between the DM-hTAU groups: *p < 0.05, **p < 0.01. *n* = 3–6 mice per group. Data are presented as mean ± s.e.m. (**I**) Pearson correlation coefficient test between the neuronal survival in the female’s subiculum and the T maze score of each of them revealed a significant linear correlation. r_(Pearson)_ = 0.6697, ***p < 0.001

Having confirmed the selectivity of αCCR2 and ruled out its non-specific effect on cognition, we next tested in DM-hTAU mice, an animal model of tauopathy, whether blocking of CCR2 would abrogate the beneficial response of αPD-L1 treatment, and if so, which additional cells beyond the monocytes are involved in such a therapeutic effect. To this end, we administered αCCR2 in a protocol that blocks the monocytes at the time of αPD-L1 injection, and for the first 2 weeks thereafter (Fig. 2A), the period considered as critical for their recruitment into the brain following the treatment [11, 19]. We compared the cognitive performance of DM-hTAU mice treated with αPD-L1 alone, to that of αCCR2 + αPD-L1-treated group. The experimental design also included a group of DM-hTAU mice that received control antibody injection (control IgG group), a group of DM-hTAU mice that received only the αCCR2 injections (αCCR2 group), to determine possible effects of monocyte blockade on cognitive behavior and pathology, and WT mice that were not treated and served as healthy controls. Four weeks after αPD-L1 treatment, mice were tested by a battery of cognitive procedures including novel arm exploration test in a T-maze, spontaneous alternation task in a Y-maze, and NOR paradigm. Brains were excised for measurement of pathological tau and neuronal survival (Fig. 2A).

Testing the mice by T-maze revealed that αPD-L1 treatment resulted in a significant improvement in exploration of the novel arm, relative to the control IgG-injected group (Fig. 2B). In addition, assessment of the mice in the Y-maze showed a significant increase in spontaneous alternation behavior of the αPD-L1-treated animals (Fig. 2C). Similarly, αPD-L1-treated mice exhibited significantly higher exploration time of the novel object in the NOR test (Fig. 2D). When monocytes were blocked by αCCR2 injections, the beneficial response of αPD-L1 on short-term memory, alternation behavior and recognition memory was abrogated in all three tasks (αCCR2 + αPD-L1 versus αPD-L1 groups; Fig. 2B–D). Administration of αCCR2 alone compared to the control IgG-injected group showed no effect on the performance of DM-hTAU mice in any of the three tasks examined (Fig. 2B–D). Assessment of the general locomotor activity did not show any changes between the experimental groups (Additional file 2). Thus, the beneficial effect of αPD-L1 treatment on brain function was abrogated when CCR2 was blocked.

Since the expression level of the mutated human tau in this model is relatively low (5–10%) [28], we tested in an independent experiment whether we could detect hyperphosphorylation of human tau by ELISA, and if so, whether an effect of αPD-L1 would be evident. To this end, we measured hippocampal human tau pS199 using a commercially available ELISA kit (see Methods), 1 month following αPD-L1 treatment, and compared αPD-L1 and IgG-injected groups. Untreated WT mice served as healthy controls (Additional file 3). The results obtained showed a very low level of human tau pS199 in the IgG-injected DM-hTAU group. The WT group showed a negligible level (Additional file 3). Investigating the effect of αPD-L1, we found a small, but significant reduction of ~ 13% in human tau pS199 compared to the IgG-injected group (Additional file 3). However, since the measured human tau pS199 levels were very low, making quantification difficult, we decided to evaluate the brain pathology of the animals that underwent behavioral testing using HTRF immunoassay (see Methods), which is known to be highly sensitive [46]. Specifically, following completion of behavioral studies, aggregated human tau protein levels were measured in cortices using a commercially available kit (Fig. 2E; see Methods). One month after αPD-L1 treatment, cortices derived from DM-hTAU mice showed significantly reduced aggregated human tau levels, as compared to the IgG-treated group, whereas αCCR2 prevented this beneficial response. Administration of αCCR2 by itself (αCCR2 group) did not cause any increase in aggregated human tau load, in comparison to the IgG-treated group (Fig. 2E). Furthermore, analysis of a possible association between aggregated human tau levels and cognitive performance, revealed a significant negative linear correlation between the amount of cortical aggregated human tau levels and the percentage of exploration time of the novel arm in the T-maze (Fig. 2F), suggesting that improved cognitive performance is associated with reduced aggregated human tau load.

To address the effect of the treatment on neuronal survival, we counted pyramidal neurons in the subiculum using Cresyl violet staining (Fig. 2G, H). We detected a reduction in the number of cells in brain sections derived from IgG- injected DM-hTAU mice, relative to the WT group. αPD-L1 treatment resulted in a significant improvement in neuronal survival, while αCCR2 abrogated this beneficial effect (Fig. 2H). Furthermore, we found a significant correlation between the neuronal survival in the subiculum and the percentage of exploration time of the novel arm in the T-maze (Fig. 2I), indicating that improved cognitive performance is associated with enhanced neuronal survival.

Overall, these results clearly demonstrate that blocking CCR2 abrogated the beneficial effect of αPD-L1 on cognitive behavior, tau pathology and neuronal survival in DM-hTAU mice.

### Upregulation of circulating CCR2^+^ monocytes and FOXP3^+^ regulatory CD4^+^ T cells following anti-PD-L1 antibody immunotherapy

**Fig. 3:**
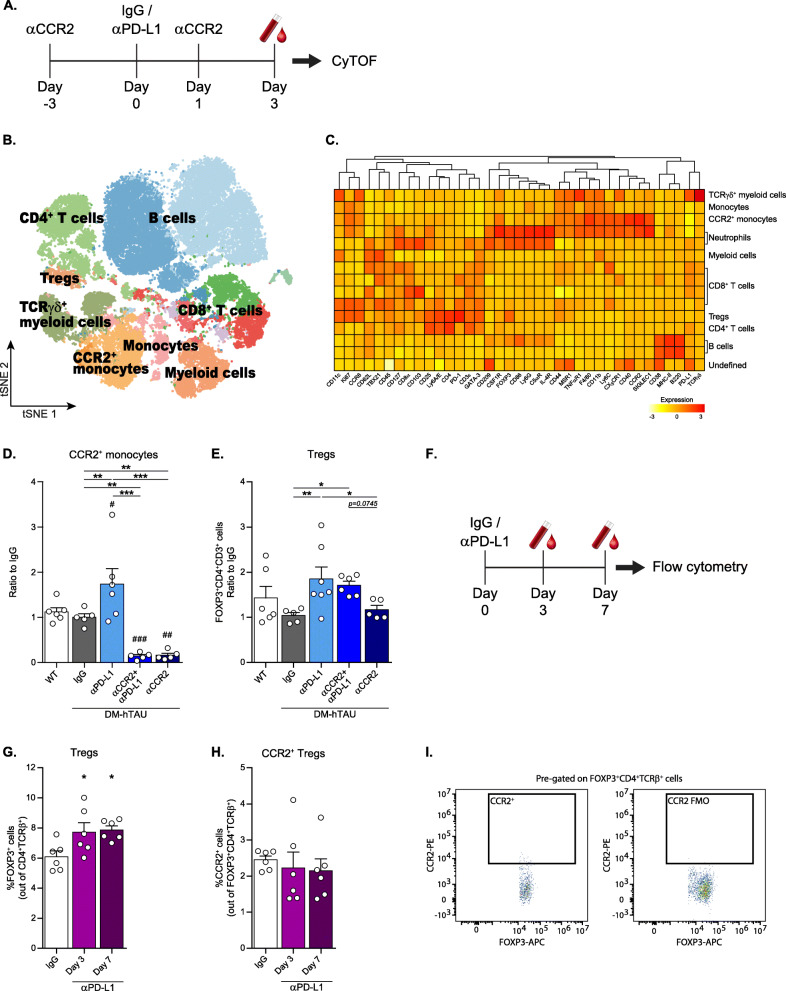
Upregulation of circulating CCR2^+^ monocytes and FOXP3^+^ regulatory CD4^+^ T cells following anti-PD-L1 antibody immunotherapy. This experiment included four groups of DM-hTAU mice treated either with: IgG, αPD-L1, αCCR2 + αPD-L1, or αCCR2. An additional group of WT mice served as healthy controls. **(A**) Schematic presentation of experimental design: αCCR2 was i.p. injected to DM-hTAU mice 3 days prior (Day − 3) to αPD-L1 or IgG (Day 0), and again 1 day after αPD-L1 treatment (Day 1). Blood was sampled 3 days following αPD-L1 treatment and analyzed by CyTOF. (**B**) FlowSOM clustering over tSNE plot showing different immune cell populations. (**C**) Heatmap of the CyTOF data showing Z-score of mean expression levels of the different markers across distinct CD45^+^ immune cell populations. (**B, C**) Representative results of one of three independent experiments. (**D**) Quantification of CCR2^+^ monocytes as measured by CyTOF. The percentage of the entire cell population per mouse is presented relative to the control IgG group. One-way ANOVA F_(4,22)_ = 14.14, ***p < 0.0001. *Post-hoc* uncorrected Fisher’s LSD multiple comparisons between DM-hTAU groups and WT: #*p* < 0.05, ##*p* < 0.01, ###*p* < 0.001. *Post-hoc* uncorrected Fisher’s LSD multiple comparisons between the DM-hTAU groups: **p < 0.01, ***p < 0.001. (**E**) Quantification of Tregs as determined by CyTOF and analyzed by manual gating. The abundance of each cell population per mouse is presented as the ratio relative to the control IgG group. One-way ANOVA F_(4,22)_ = 2.986, *p = 0.0392. *Post-hoc* uncorrected Fisher’s LSD multiple comparisons between the DM-hTAU groups: *p < 0.05, **p < 0.01. *n* = 5–6 mice per group. Data are presented as mean ± s.e.m. (**F**) WT mice were injected with αPD-L1, and after 3 and 7 days, blood was sampled and analyzed by multiparametric flow cytometry. (**G**) Flow cytometry analysis of blood Tregs (One-way ANOVA F_(2,15)_ = 4.762, *p = 0.025. *Post-hoc* uncorrected Fisher’s LSD multiple comparisons between αPD-L1 to IgG groups: *p < 0.05). (**H**) Flow cytometry analysis of CCR2 expression by blood Tregs, and (**I**) representative dot plot of the gating strategy. Splenocytes were used as FMO; n = 6 mice per group. Data are presented as mean ± s.e.m.

Since PD-1/PD-L1 blockade unleashes various immune cell types from inhibition [47], we next analyzed the blood after αPD-L1 injection to detect changes in monocytes, and in other immune cells, such as T cells, that might subsequently take part in the repair process. For this purpose, we used Cytometry by time of flight (CyTOF), which allows the simultaneous measurement of dozens of surface markers expressed by the cells [48, 49], to characterize the immune cell populations in peripheral blood. Specifically, based on past experience, we analyzed the blood immune profile of DM-hTAU mice 3 days after the treatment. We compared αPD-L1, IgG and αCCR2 + αPD-L1 injected groups (Fig. 3A). A group of DM-hTAU mice that received only αCCR2 injections (αCCR2 group) was included to evaluate possible systemic effects of the injected antibody on the immune cell populations. WT mice that were not treated served as a reference for the healthy blood immune profile. Analysis of the data obtained by CyTOF using FlowSOM algorithm (see Methods), identified clusters of distinct subsets of cells based on their surface and intracellular marker expression (Fig. 3B, C). Comparison of the percentage of cells per cluster across the different conditions identified a unique population of CCR2^+^ monocytes that was significantly upregulated following αPD-L1 treatment, in comparison to the IgG-injected group, and was abrogated upon αCCR2 administration (Fig. 3D). This population was identified as CD45^+^ CD11b^hi^ CD44^+^ CCR2^+^ CCR6^+^ Ly6C^hi^ F4/80^+^ TNFαRI^+^ CX3CR1^+^ Ki67^+^ (Fig. 3C), a profile characteristic of trafficking monocytes [36, 50]. This population could also express IL-4R, MSR1, SIGLEC1 and CD11c (Fig. 3C), a phenotype reminiscent of the monocyte-derived macrophages identified in the brain in our previous study [19].

Using a manual gating strategy, which allowed detection of cell populations with lower prevalence, we further observed a significant increase in the number of circulating Tregs following αPD-L1 treatment, in comparison to the IgG-injected group. The abundance of this population in the blood was not reduced by αCCR2 injections (Fig. 3E). FlowSOM algorithm analyses did not show any effect of αPD-L1 treatment on the levels other immune cell types detected (Fig. 3B, C). To test whether the elevation of Tregs following PD-L1 blockade is observed in healthy animals as well, we injected WT mice with αPD-L1 (or IgG as a control) and assessed peripheral CD4^+^ T cell populations 3 and 7 days after the treatment, using multiparametric flow cytometry (Fig. 3F). We found a significant increase in Tregs after αPD-L1 administration, as compared to the control IgG-treated group (Fig. 3G). Analysis of CCR2 expression by these Tregs showed that only a minor percentage of the cells [2.274 ± 0.1776% (mean ± s.e.m)] expressed this marker (Fig. 3H, I). Similar results were observed in T cells obtained from the spleen, which also exhibited a significant upregulation of memory CD4^+^ T cells (Additional file 4 A-C). The lack of expression of CCR2 by T cell populations is consistent with the findings described above, showing that systemic CCR2 blockade had no effect on the blood levels of total CD4^+^ T cells (Fig. 1) and of Tregs (Figs. 1, 3E).

### CCR2 blockade prevents accumulation of FOXP3^+^ regulatory CD4^+^ T cells in DM-hTAU brains treated with anti-PD-L1 antibody

**Fig. 4:**
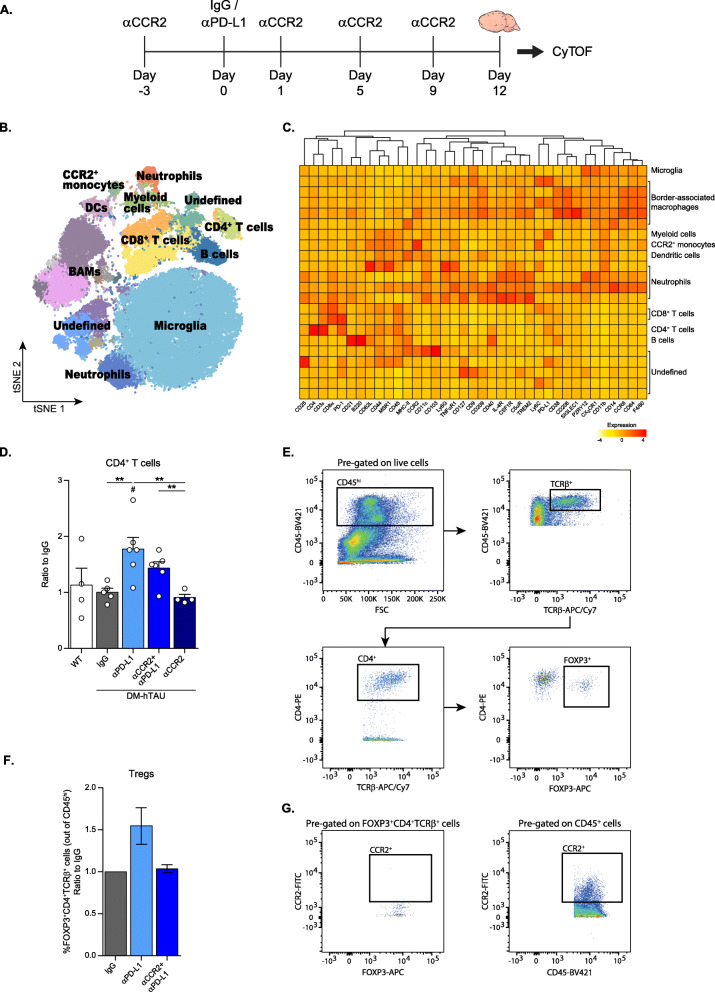
CCR2 blockade prevents accumulation of FOXP3^+^ regulatory CD4^+^ T cells in DM-hTAU brains treated with anti-PD-L1. This experiment included four groups of DM-hTAU mice treated either with: IgG, αPD-L1, αCCR2 + αPD-L1, or αCCR2. An additional group of WT mice served as healthy controls. (**A**) Schematic presentation of experimental design: αCCR2 was i.p. injected to DM-hTAU mice 3 days prior (Day − 3) to αPD-L1 or IgG (Day 0), and again on days 1, 5 and 9. The brains were analyzed by CyTOF 3 days after the last αCCR2 injection (Day 12). (**B**) FlowSOM clustering over tSNE plot showing different immune populations. DCs- dendritic cells, BAMs- border associated macrophages. (**C**) Heatmap of the CyTOF data showing Z-score of mean expression levels of the different markers across distinct CD45^+^ immune cell populations. (**B, C**) Representative results from one of three independent experiments. (**D**) Quantification of CD4^+^ T cells as measured by CyTOF. The percentage of each cell population per mouse was calculated, and normalized to the control IgG group. (One-way ANOVA F_(4,20)_ = 4.427, *p = 0.01. *Post-hoc* uncorrected Fisher’s LSD multiple comparisons between DM-hTAU groups to WT: #*p* < 0.05. *Post-hoc* uncorrected Fisher’s LSD multiple comparisons between the DM-hTAU groups: **p < 0.01). *n* = 4–6 mice per group. Data are presented as mean ± s.e.m. (**E**) Flow cytometry gating strategy of Tregs in the brain of DM-hTAU mice. (**F**) Flow cytometry analysis of Tregs obtained from two experiments, in which each group was comprised of a pool of 10 mice. The results are presented as a ratio to the control IgG group. Data are presented as mean ± s.e.m. (**G**) Representative flow cytometry dot plots demonstrating negligible expression of CCR2 by the Tregs detected in the brains

In acute CNS insults, T cells and primarily Tregs, were found to be important for repair, in addition to monocytes, and their presence was detected at late time points in the healing process [8, 9]. We therefore hypothesized that disease modification triggered by αPD-L1 might also involve CD4^+^ T cell populations within the brain. We used CyTOF to obtain an unbiased comparison of the cellular composition of the brains of DM-hTAU mice following αPD-L1 administration, versus control IgG- and αCCR2 + αPD-L1- injected groups (Fig. 4A). Moreover, a group of DM-hTAU mice injected only with αCCR2, and a group of WT mice were used as controls. All groups were tested 12 days after αPD-L1 administration, to allow detection of potential late cell recruitment. Due to technical limitations in the number of cells obtained per brain and the cell loss involved in the intracellular staining procedure, we assessed only extracellular markers. Analysis of the data acquired by CyTOF using FlowSOM algorithm, classified different subsets of cells (Fig. 4B, C). Evaluation of the different myeloid cells, including the resident microglia and border-associated macrophages (BAMs) (Fig. 4B, C), did not reveal any changes in their phenotype under any of the experimental conditions. Next, we examined the CD4^+^ T cell population by comparing the percentage of cells in the clusters across the different conditions. We found a significant increase in CD4^+^ T cells following αPD-L1 treatment, relative to the IgG-treated group. Injections of αCCR2 resulted in a small, but not significant, reduction in the number of CD4^+^ T cells in the group that was treated with both αPD-L1 and αCCR2 (Fig. 4D). Analyses of other immune cell populations did not show any effect for αPD-L1 treatment. We considered that by using CyTOF without intracellular staining, we could miss a sub-population of CD4^+^ T cells, such as Tregs, that might be indirectly affected by CCR2 blockade. Therefore, we conducted an experiment in which we used multiparametric flow cytometry to study this population. Specifically, we quantified FOXP3^+^ CD4^+^ TCRβ^+^ cells in the brain, and compared αPD-L1 to IgG and to αCCR2 + αPD-L1-injected groups (Fig. 4E, F). Since we expected only a small number of such cells in the brain, we pooled 10 mice per condition. Our results revealed that αPD-L1 treatment led to an increased number of Tregs in the brain in comparison to the IgG-injected group. Injections of αCCR2 resulted in a complete elimination of the accumulated Tregs in the brain (Fig. 4F). Importantly, these accumulated Tregs were found to express only negligible levels of CCR2 (Fig. 4G), indicating that their accumulation in the brain was not directly mediated through CCR2 itself. Of note, although 12 days following αPD-L1 treatment is a relatively late time point for the detection of an increase in infiltrating monocytes, the reduction in Treg numbers due to CCR2 blockade (Fig. 4F) was proportional to the induced reduction of monocytes at this time point (Additional files 5, 6). Together, these findings suggest that although Tregs do not express CCR2, their accumulation in the diseased brain is affected by CCR2^+^ monocytes.

### Association between the effect of anti-PD-L1 antibody treatment on inflammation and the T-cell chemoattractant *Cxcl12*

**Fig. 5:**
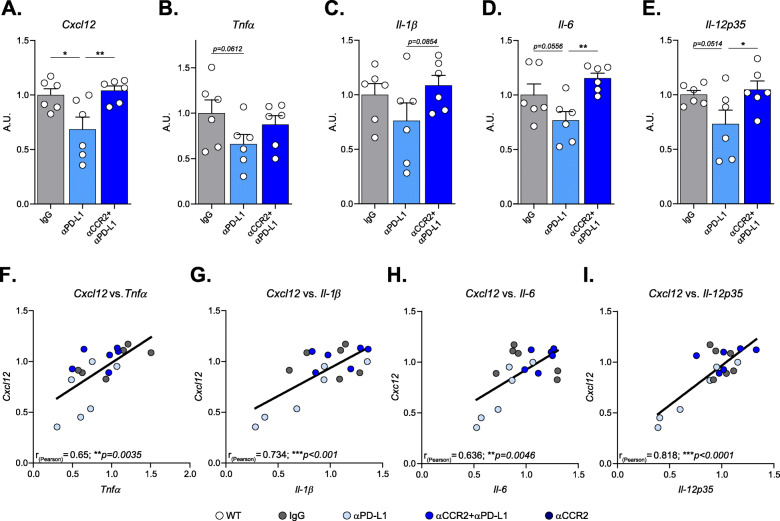
Anti-PD-L1 antibody therapeutic effect on inflammation reduction is associated with the T-cell chemoattractant *Cxcl12*. This experiment included three groups of DM-hTAU mice treated either with: IgG, αPD-L1, or αCCR2 + αPD-L1. αCCR2 was i.p. injected to DM-hTAU mice 3 days prior (Day − 3) to αPD-L1 (Day 0), and again on days 1, 5 and 9. Hippocampi were excised and analyzed by RT-qPCR 3 days after the last αCCR2 injection (Day 12). (**A**) RT-qPCR results for *Cxcl12* (One-way ANOVA F_(2,15)_ = 6.3419, **p = 0.0097), (**B**) *Tnfα* (One-way ANOVA F_(2,15)_ = 2.095, *p* = 0.1576), (**C**) *Il-1β* (One-way ANOVA F_(2,15)_ = 1.821, *p* = 0.1959), (**D**) *Il-6* (One-way ANOVA F_(2,15)_ = 5.958, *p = 0.0125), and (**E**) *Il-12p35* (One-way ANOVA F_(2,15)_ = 3.607, *p* = 0.0526). (**A-E**) *Post-hoc* uncorrected Fisher’s LSD multiple comparisons between the groups: *p < 0.05, **p < 0.01; n = 6 mice per group. Data are presented as mean ± s.e.m. (**F**) Pearson correlation coefficient tests revealed significant linear correlations between the measured mRNA levels of *Cxcl12* and of the inflammatory cytokines: *Tnfα* (r_(Pearson)_ = 0.65, **p = 0.0035), (**G**) *Il-1β* (r_(Pearson)_ = 0.734, ***p < 0.001), (**H**) *Il-6* (r_(Pearson)_ = 0.636, **p = 0.0046), and (**I**) *Il-12p35* (r_(Pearson)_ = 0.818, ***p < 0.0001)

Next, we examined whether T cell chemoattractants might be involved in Treg accumulation in the brain. To this end, we measured mRNA expression levels by RT-qPCR in hippocampi dissected from brains of αPD-L1, IgG and αCCR2 + αPD-L1-injected groups, 12 days following the αPD-L1 treatment. We found a significant reduction in *Cxcl12* level following αPD-L1 treatment, compared to the IgG-injected group, and this effect was eliminated when CCR2 was blocked (Fig. 5A).

Next we tested whether the inflammatory state within the brain would be modified following the treatment. To this end, we further measured a panel of inflammatory cytokines. Analyses of Tumor necrosis factor alpha *(Tnfa),* Interleukin-1 beta *(Il-1β),* Interleukin-6 *(Il-6)* and Interleukin-12 subunit alpha (*Il-12p35)* revealed a reduction in their expression levels following αPD-L1 treatment, compared to the IgG-injected group (Fig. 5B–E). This beneficial effect was reduced (Fig. 5B, C), and even abrogated (Fig. 5D, E) when αCCR2 was administered. Moreover, evaluation of the association between *Cxcl12* and the inflammatory cytokines revealed significant correlations (Fig. 5F–I), suggesting that, in addition to monocyte-derived macrophages, the accumulated Tregs in the brain could participate in reduction of inflammation, which is accompanied by a decrease in distress signals secreted by the tissue.

## Discussion

This study describes the role of the CCR2/CCL2-axis in the communication between the brain and the immune system to combat dementia. Using a mouse model of tauopathy, we applied a therapeutic approach involving activation of the immune system by blockade of the PD-1/PD-L1 inhibitory pathway, which was previously shown to be beneficial in modifying AD and tauopathy [11, 19]. Blocking monocytes, using a CCR2-specific antibody, abrogated the beneficial effect of αPD-L1 treatment on cognitive performance, brain pathology, local inflammation, and neuronal survival. Moreover, we discovered that CCR2-expressing monocytes, beyond their known direct functions mediating removal of neurotoxic molecules and controlling local inflammation [14, 19–22], are also affecting the accumulation of Tregs in the brain.

Wound healing in tissues outside the brain is comprised of several steps that involve the immune system. Activated monocytes, which are known to express high levels of Ly6C and CCR2, migrate to the pathological site within 48–96 h post-injury [36, 51–54], while elevation in their number can be detected in the peripheral blood [55, 56]. Here, we used systemic αPD-L1 administration to unleash the immune system, which resulted in an increase of circulating CCR2^+^ Ly6C^hi^ monocytes, 3 days after the treatment, suggesting immune activation reminiscent of the physiological response following injury. Moreover, we detected an elevation of systemic Tregs following αPD-L1 immunotherapy, in accordance with several studies demonstrating an increase in their numbers following PD-1/PD-L1 blockade [57–60]. In addition, in the present study, we demonstrate that αPD-L1 led to the accumulation of CD4^+^ T cells and Tregs in the brain, further supporting a sequence of events similar to that observed in CNS repair processes [8, 9, 61–65].

Monocyte-derived macrophages were found to play diverse roles in various CNS pathologies [66, 67]. Following acute CNS insults, they produce inflammatory (e.g. IL-1β and TNFα) [64] and anti-inflammatory cytokines (e.g. IL-10 and TGFβ) [39, 64, 68], secrete matrix-degrading enzymes (e.g. MMP13) [68], and phagocytose damage-associated molecular patterns via scavenger receptors, such as MSR1 and MARCO [69]. In animal models of AD, monocyte-derived macrophages have been shown to play a major role in disease mitigation [70–72]. As such, they were shown to phagocytose amyloid-beta (Aβ) plaques [14, 20] and to reduce the levels of soluble Aβ oligomers [21, 22], in part via expression of MSR1 [23]. Specifically, in the same mouse model of tauopathy that we used here, MSR1 expressed by monocyte-derived macrophages was found to be crucial for the beneficial response to PD-L1 immunotherapy [19]. Thus, the observed reduction of the parenchymal inflammatory cytokines following treatment with αPD-L1, found in the present study, could be due to the contribution of the recruited monocyte-derived macrophages presenting an M2-like profile, as previously described [19]. Importantly, while the role of the resident microglia in these pathologies is still unclear [73, 74], macrophages were demonstrated as essential players in both amyloidosis and tauopathies, as is also shown in the present study.

Of note, the CCR2/CCL2 axis was demonstrated as beneficial in animal models of AD. As such, mouse models of amyloidosis deficient in CCR2 exhibited exacerbation of cognitive decline and amyloid pathology [21, 75, 76]; however, the role of CCR2/CCL2 axis in tauopathy was never substantiated. In the current study, we found that blocking CCR2 not only abrogated the beneficial effect of PD-L1 immunotherapy on cognitive performance, local brain inflammation, and pathological manifestations, but also prevented the accumulation of Tregs in the diseased brain. Notably, since Treg levels in the blood were not reduced by αCCR2, it is likely that Treg accumulation in the brain is indirectly controlled by this axis. The accumulated Tregs could be an outcome of homing to the brain induced by monocytes [8], and their subsequent local proliferation [9, 77, 78]. Alternatively, the increased Tregs might result from local conversion of infiltrating CD4^+^ T cells [79], or expansion of brain-resident Tregs [80] induced by the parenchymal milieu that was modified by the monocytes. Our results are in line with previous findings in spinal cord injury [8] and in mouse models of experimental autoimmune uveitis [40], identifying macrophages as modulators of Treg accumulation at the pathological site. Although neither flow cytometry nor CyTOF experiments can determine the localization in the brain of either the macrophages or of the Tregs, our previous publication demonstrated the presence of monocyte-derived macrophages within the parenchyma following αPD-L1 treatment [19]. Yet, we cannot rule out the possibility that some of the cells that home to the brain, reside in its borders, including the meninges, CP and perivascular spaces, and exert their activity from these compartments [81–83].

Within the CNS, Tregs were found to play important roles under diverse pathological conditions. In acute conditions, such as in spinal cord injury, Tregs are recruited to the lesion site by monocyte-derived macrophages, and act as anti-inflammatory cells [8]. In acute brain pathologies, such as ischemic stroke and hemorrhage, Tregs were found to accumulate in the parenchyma, to suppress astrogliosis and potentiate neurological recovery, limit inflammatory cytokine production, reduce infiltration of leukocytes into the brain, and to locally modulate the phenotype of immune cells towards an anti-inflammatory profile [9, 65, 84].

In chronic neurodegenerative conditions, Tregs were shown to play a beneficial role as well [24, 85]. For example, in an animal model of Amyotrophic lateral sclerosis (ALS), Tregs were found to be increased within the CNS during the early slowly progressing stages, but decreased when disease progression accelerated. The CNS Tregs secreted anti-inflammatory cytokines and modulated microglial activity, and were suggested to augment neuroprotection and to prolong survival [86]. Additionally, systemic immune activation in ALS mice by myelin-derived antigen led to accumulation of Tregs in the CNS alongside monocyte recruitment, and together, these immune cells induced a shift towards an anti-inflammatory milieu and slowed disease progression [22]. In an animal model of Parkinson’s disease, adoptive transfer of Tregs led to their accumulation in the substantia nigra, prevented dopaminergic neuronal loss, improved functional recovery, and attenuated the inflammatory reaction in the brain. Using in-vitro paradigms, it was demonstrated that the Treg-induced neuroprotective effect occurred through a mechanism requiring cell-to-cell contact [85]. In the current study, the accumulation of Tregs observed in the diseased neurodegenerative brain, as part of the therapeutic cascade of immunological events induced by αPD-L1, may play a pivotal role in modulating inflammation and supporting neuroprotection within the brain tissue.

Interestingly, blocking the PD-1/PD-L1 pathway was demonstrated to be beneficial in other brain pathologies, such as Progressive multifocal leukoencephalopathy in humans [87], and ischemic stroke in mice [88]. In both studies, the researchers found an improvement in neurological and pathological symptoms accompanied by changes in activation of immune cells. In the stroke model, enhanced accumulation of regulatory CD8^+^ T cells and a shift towards an anti-inflammatory milieu in the brain was found, as well [88]. 

Overall, our results emphasize that disease modification in tauopathy, which occurs in response to PD-L1 immunotherapy, resembles the immunological sequence of events comprising a natural healing process. These observations also substantiate the contention that targeting the peripheral immune system to treat neurodegenerative disease by αPD-L1, harnesses the body’s own mechanism of repair. Furthermore, the complete abrogation of the effect of αPD-L1 on cognitive performance and disease pathology following monocyte blockade, supports the notion that the primary effect of αPD-L1 occurs outside the brain, and thereafter, its effects within the brain are mediated by cellular, and possibly, by soluble messengers.

## Conclusions

Taken together, our results highlight a general mechanism that is needed to combat dementia, in which the CCR2/CCL2 axis, together with additional chemokines, such as *Cxcl12*, regulate the communication pathway between the brain and the immune system. Blood-derived monocytes play an essential role in this interaction. Our results therefore suggest that this immunotherapy approach could also be broadly applicable to treatment of dementia of various origins, regardless of the pathophysiology.

## Supplementary Information

**Additional file 1 Supplementary Table 1.** Antibodies used in CyTOF experiments.

**Additional file 2 Supplementary Fig. 1. Lack of changes in general locomotor activity following anti-PD-L1 antibody or anti-CCR2 antibody injections.** This experiment included four groups of DM-hTAU mice treated either with: IgG, αPD-L1, αCCR2 + αPD-L1, or αCCR2. An additional group of WT mice served as healthy controls. αCCR2 was i.p. injected to DM-hTAU mice 3 days prior (Day − 3) to αPD-L1 or IgG (Day 0), and again on days 1, 5 and 9. The graph shows locomotor activity of females measured during the habituation trial in the novel object recognition test assessed 1 month after αPD-L1 treatment. *n* = 3–8 mice per group. Data are presented as mean ± s.e.m.

**Additional file 3 Supplementary Fig. 2. Hippocampal human tau pS199 levels are reduced following anti-PD-L1 antibody treatment.** αPD-L1 or IgG were i.p. injected to DM-hTAU mice. Hippocampi were analyzed for human tau pS199 levels 1 month afterwards. Untreated WT mice served as healthy controls. The graph shows hippocampal human tau protein pS199 amounts (pg) that were measured by ELISA and were normalized to the amount of total protein in each tissue (μg). One-way ANOVA F_(2,15)_ = 135.2, ***p < 0.0001. *Post-hoc* uncorrected Fisher’s LSD multiple comparisons between DM-hTAU groups to WT: ###*p* < 0.001. *Post-hoc* uncorrected Fisher’s LSD multiple comparisons between the DM-hTAU groups: *p < 0.05. *n* = 6 mice per group. Data are presented as mean ± s.e.m.

**Additional file 4 Supplementary Fig. 3. WT spleens show changes in CD4**^**+**^ **T cell populations following anti-PD-L1 antibody treatment.** WT mice were i.p. injected with αPD-L1 (or IgG), and spleens were collected 3 and 7 days after the treatment and analyzed by multiparametric flow cytometry**.** (**A**) Flow cytometry analyses of Tregs (One-way ANOVA F_(2,15)_ = 7.697, **p = 0.005. *Post-hoc* uncorrected Fisher’s LSD multiple comparisons between αPD-L1 and IgG groups: *p < 0.05, **p < 0.01) and (**B**) their CCR2 expression. (**C**) Flow cytometry analysis of memory CD4^+^ T cells (One-way ANOVA F_(2,15)_ = 4.374, *p = 0.0319. *Post-hoc* uncorrected Fisher’s LSD multiple comparisons between αPD-L1 to IgG groups: *p < 0.05). n = 6 mice per group. Data are presented as mean ± s.e.m.

**Additional file 5 Supplementary Fig. 4. Anti-CCR2 antibody induces reduction in brain infiltrating monocytes following anti-PD-L1 antibody treatment.** This experiment included three groups of DM-hTAU mice treated either with: IgG, αPD-L1 or αCCR2 + αPD-L1. αCCR2 was i.p. injected to DM-hTAU mice 3 days prior (Day − 3) to αPD-L1 (Day 0), and then again on days 1, 5 and 9. The brains were analyzed by multiparametric flow cytometry 3 days after the last αCCR2 injection (Day 12). (**A**) Flow cytometry gating strategy, and (**B**) quantification of infiltrating monocytes in the brain of DM-hTAU mice. One-way ANOVA F_(2,15)_ = 3.591, *p* = 0.0532. *Post-hoc* uncorrected Fisher’s LSD multiple comparisons between the groups: *p < 0.05. n = 6 mice per group. Data are presented as mean ± s.e.m.

**Additional file 6 Supplementary Table 2.** The ratio between the number of cells found in the brain following co-administration of αCCR2 and αPD-L1 relative to administration of αPD-L1 alone, was similar for both monocytes and Tregs.

## Data Availability

The data that support the findings of this study are available from the corresponding author upon reasonable request.

## Abbreviations

Aβ: Amyloid-beta
AD: Alzheimer’s disease
ALS: Amyotrophic lateral sclerosis
CNS: Central nervous system
CyTOF: Cytometry by Time OF Flight
ELISA: Enzyme-linked immunosorbent assay
FRET: Fluorescence resonance energy transfer
HTRF: Homogeneous Time Resolved Fluorescence
i.p.: Intraperitoneally
ITI: Inter-trial interval
NOR: Novel object recognition
Tregs: FOXP3+ regulatory CD4+ T cells
WT: Wild-type
αCCR2: Anti-CCR2 antibody
αPD-L1: Anti-PD-L1 antibody
