# Supplementary material for: Key role of the CCR2-CCL2 axis in disease modification in a mouse model of tauopathy
Source: Mol Neurodegener. 2021 Jun 25;16:39. doi: 10.1186/s13024-021-00458-z (PMC8234631; doi:10.1186/s13024-021-00458-z)
Supplement: Supplementary file 1 — Additional file 1 Supplementary Table 1. Antibodies used in CyTOF experiments. [file 13024_2021_458_MOESM1_ESM.pdf]

Additional file 1 Supplementary Table 1

Antibodies used in CyTOF experiments.

Antibody	Conjugated Metal	Manufacturer	Conjugation kit
CR5a	139La	R&D; MAB6467	Lightning-Link® 139La Antibody Labeling Kit; M139-0100
Ly6G	141Pr	Fluidigm; 3141008B	-
CD86	142Nd	BioLegend; 105002	Maxpar® X8 Antibody Labeling Kit, 142Nd; 201142A
CD11c	142Nd	Fluidigm; 3142003B	-
IL-4Rα	143Nd	BioLegend; 144802	Maxpar® X8 Antibody Labeling Kit, 143Nd; 201143A
CD115	144Nd	Fluidigm; 3144012B	-
CD4	145Nd	Fluidigm; 3145002B	-
CD8α	146Nd	Fluidigm; 3146003B	-
CD103	147Sm	R&D; AF1990	Maxpar® X8 Antibody Labeling Kit, 147Sm; 201147A
CD11b	148Nd	Fluidigm; 3148003B	-
TNFαR1	149Sm	R&D; AF-425-PB	Maxpar® X8 Antibody Labeling Kit, 149Sm; 201149A
Ly6C	150Nd	Fluidigm; 3150010B	-
CD25	151Eu	Fluidigm; 3151007B	-
CD64	151Eu	Fluidigm; 3151012B	-
CD3ε	152Sm	Fluidigm; 3152004B	-
PD-L1	153Eu	Fluidigm; 3153016B	-
TER119	154Sm	Fluidigm; 3154005B	-
IL-7R	155Gd	BioLegend; 135029	Maxpar® X8 Antibody Labeling Kit, 155Gd; 201155A
PD-1	156Gd	BioLegend; 114102	Maxpar® X8 Antibody Labeling Kit, 156Gd; 201156A
CD14	156Gd	Fluidigm; 3156009B	-
FOXP3	158Gd	Fluidigm; 3158003B	-

CD9	158Gd	Fluidigm; 3158009B	-
PD-1	159Tb	Fluidigm; 3159024B	-
GATA3	159Tb	R&D; MAB6330	Maxpar® X8 Antibody Labeling Kit, 159Tb; 201159A
TBX21	160Gd	Fluidigm; 3160010B	-
P2RY12	160Gd	BioLegend; 848002	Maxpar® X8 Antibody Labeling Kit, 160Gd; 201160A
CD11c	161Dy	BioLegend; 117341	Maxpar® X8 Antibody Labeling Kit, 161Dy; 201161A
CD40	161Dy	Fluidigm; 3161020B	-
KI67	162Dy	Fluidigm; 3162012B	-
TREM2	162Dy	R&D; MAB17291	Maxpar® X8 Antibody Labeling Kit, 162Dy; 201162A
CCR6	163Dy	BioLegend; 129802	Maxpar® X8 Antibody Labeling Kit, 163Dy; 201163A
Ly6A/E	164Dy	Fluidigm; 3164005B	-
CX3CR1	164Dy	Fluidigm; 3164023B	-
TCRγδ	165Ho	BioLegend; 118101	Maxpar® X8 Antibody Labeling Kit, 165Ho; 201165A
CD25	165Ho	BioLegend; 101913	Maxpar® X8 Antibody Labeling Kit, 165Ho; 201165A
F4/80	166Er	BioLegend; 123102	Maxpar® X8 Antibody Labeling Kit, 166Er; 201166A
CCR2	167Er	R&D; MAB55381R	Maxpar® X8 Antibody Labeling Kit, 167Er; 201167A
CD40	168Er	BioLegend; 102802	Maxpar® X8 Antibody Labeling Kit, 168Er; 201168A
CR2	168Er	Fluidigm; 3168010B	-
CX3CR1	169Tm	BioLegend; 149002	Maxpar® X8 Antibody Labeling Kit, 169Tm; 201169A
CD206	169Tm	Fluidigm; 3169021B	-
SIGLEC1	170Er	Fluidigm; 3170018B	-
CD44	171Yb	Fluidigm; 3171003B	-
MSR1	172Yb	R&D; MAB1797	Maxpar® X8 Antibody Labeling Kit, 172Yb; 201172A
CD62L	173Yb	BioLegend; 104443	Maxpar® X8 Antibody Labeling Kit, 173Yb; 201173A

CD209	174Yb	R&D; MAB83451	Maxpar® X8 Antibody Labeling Kit, 174Yb; 201174A
CD38	175Lu	Fluidigm; 3175014B	-
B220	176Yb	Fluidigm; 3176002B	-
CD45	89Y	Fluidigm; 3089005B	-
MHC-II (I-A/I-E)	209Bi	Fluidigm; 3209006B	-
