# Supplementary material for: Key role of the CCR2-CCL2 axis in disease modification in a mouse model of tauopathy
Source: Mol Neurodegener. 2021 Jun 25;16:39. doi: 10.1186/s13024-021-00458-z (PMC8234631; doi:10.1186/s13024-021-00458-z)
Supplement: Supplementary file 2 — Additional file 2 Supplementary Fig. 1. Lack of changes in general locomotor activity following anti-PD-L1 antibody or anti-CCR2 antibody injections. This experiment included four groups of DM-hTAU mice treated either with: IgG, αPD-L1, αCCR2 + αPD-L1, or αCCR2. An additional group of WT mice served as healthy controls. αCCR2 was i.p. injected to DM-hTAU mice 3 days prior (Day − 3) to αPD-L1 or IgG (Day 0), and again on days 1, 5 and 9. The graph shows locomotor activity of females measured during the habituation trial in the novel object recognition test assessed 1 month after αPD-L1 treatment. n = 3–8 mice per group. Data are presented as mean ± s.e.m. [file 13024_2021_458_MOESM2_ESM.pdf]

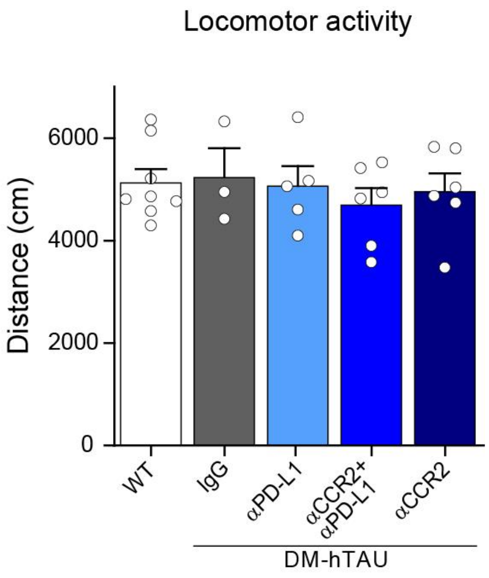

Additional file 2 Supplementary Figure 1. Lack of changes in general locomotor activity following anti-PD-L1 antibody or anti-CCR2 antibody injections. This experiment included four groups of DM-hTAU mice treated either with: IgG, αPD-L1, αCCR2+αPD-L1, or αCCR2. An additional group of WT mice served as healthy controls. αCCR2 was i.p. injected to DM-hTAU mice 3 days prior (Day -3) to αPD-L1 or IgG (Day 0), and again on days 1, 5 and 9. The graph shows locomotor activity of females measured during the habituation trial in the novel object recognition test assessed 1 month after αPD-L1 treatment. n=3-8 mice per group. Data are presented as mean ± s.e.m.
