# Supplementary material for: Key role of the CCR2-CCL2 axis in disease modification in a mouse model of tauopathy
Source: Mol Neurodegener. 2021 Jun 25;16:39. doi: 10.1186/s13024-021-00458-z (PMC8234631; doi:10.1186/s13024-021-00458-z)
Supplement: Supplementary file 3 — Additional file 3 Supplementary Fig. 2. Hippocampal human tau pS199 levels are reduced following anti-PD-L1 antibody treatment. αPD-L1 or IgG were i.p. injected to DM-hTAU mice. Hippocampi were analyzed for human tau pS199 levels 1 month afterwards. Untreated WT mice served as healthy controls. The graph shows hippocampal human tau protein pS199 amounts (pg) that were measured by ELISA and were normalized to the amount of total protein in each tissue (μg). One-way ANOVA F(2,15) = 135.2, ***p < 0.0001. Post-hoc uncorrected Fisher’s LSD multiple comparisons between DM-hTAU groups to WT: ###p < 0.001. Post-hoc uncorrected Fisher’s LSD multiple comparisons between the DM-hTAU groups: *p < 0.05. n = 6 mice per group. Data are presented as mean ± s.e.m. [file 13024_2021_458_MOESM3_ESM.pdf]

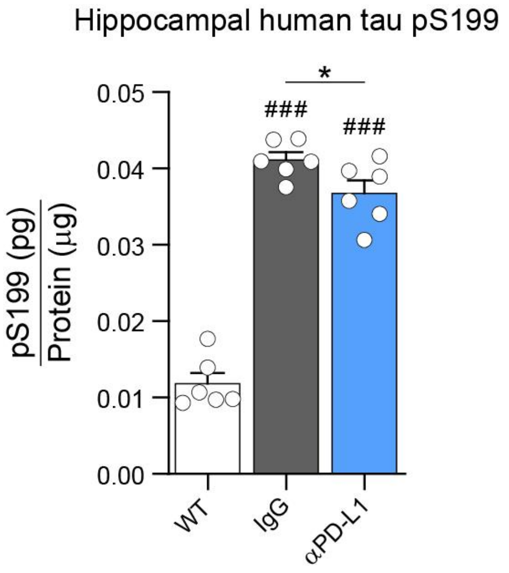

Additional file 3 Supplementary Figure 2. Hippocampal human tau pS199 levels are reduced following anti-PD-L1 antibody treatment. αPD-L1 or IgG were i.p. injected to DM-hTAU mice. Hippocampi were analyzed for human tau pS199 levels 1 month afterwards. Untreated WT mice served as healthy controls. The graph shows hippocampal human tau protein pS199 amounts (pg) that were measured by ELISA and were normalized to the amount of total protein in each tissue (µg). One-way ANOVA $F_{(2,15)}=135.2$, ***$p<0.0001$. *Post-hoc* uncorrected Fisher's LSD multiple comparisons between DM-hTAU groups to WT: ###$p<0.001$. *Post-hoc* uncorrected Fisher's LSD multiple comparisons between the DM-hTAU groups: *$p<0.05$. n=6 mice per group. Data are presented as mean ± s.e.m.
