# Supplementary material for: Key role of the CCR2-CCL2 axis in disease modification in a mouse model of tauopathy
Source: Mol Neurodegener. 2021 Jun 25;16:39. doi: 10.1186/s13024-021-00458-z (PMC8234631; doi:10.1186/s13024-021-00458-z)
Supplement: Supplementary file 4 — Additional file 4 Supplementary Fig. 3. WT spleens show changes in CD4+ T cell populations following anti-PD-L1 antibody treatment. WT mice were i.p. injected with αPD-L1 (or IgG), and spleens were collected 3 and 7 days after the treatment and analyzed by multiparametric flow cytometry. (A) Flow cytometry analyses of Tregs (One-way ANOVA F(2,15) = 7.697, **p = 0.005. Post-hoc uncorrected Fisher’s LSD multiple comparisons between αPD-L1 and IgG groups: *p < 0.05, **p < 0.01) and (B) their CCR2 expression. (C) Flow cytometry analysis of memory CD4+ T cells (One-way ANOVA F(2,15) = 4.374, *p = 0.0319. Post-hoc uncorrected Fisher’s LSD multiple comparisons between αPD-L1 to IgG groups: *p < 0.05). n = 6 mice per group. Data are presented as mean ± s.e.m. [file 13024_2021_458_MOESM4_ESM.pdf]

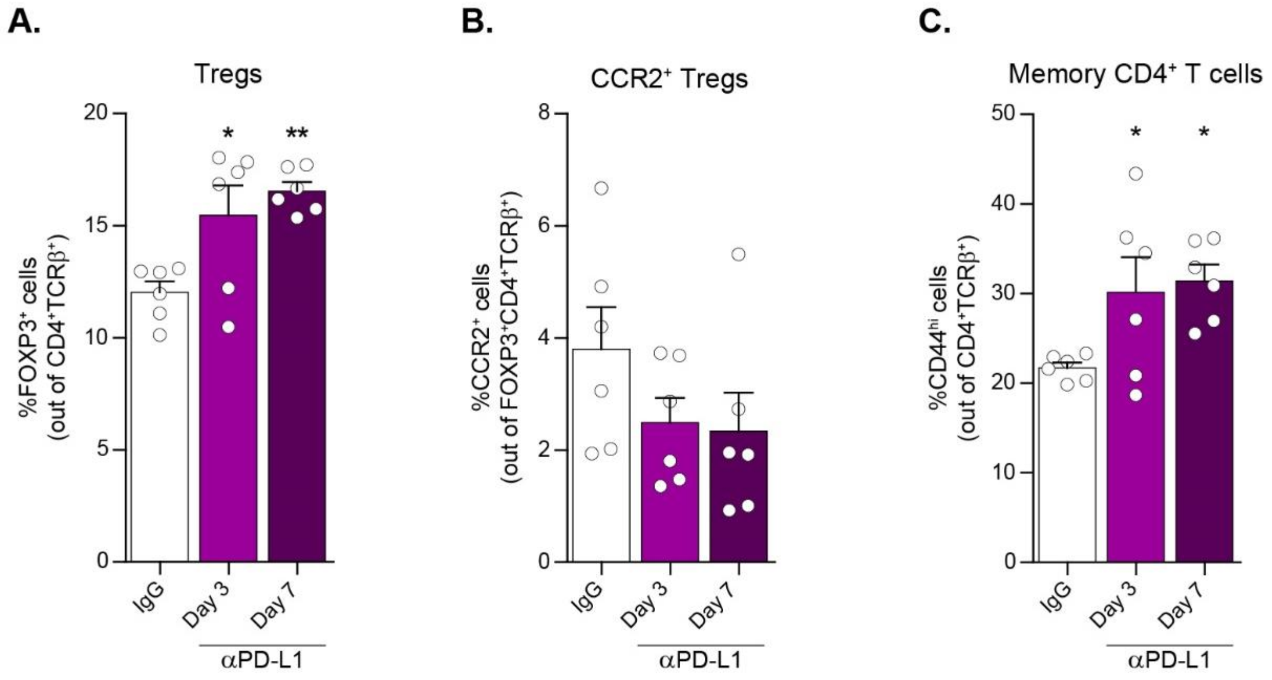

Additional file 4 Supplementary Figure 3. WT spleens show changes in CD4$^+$ T cell populations following anti-PD-L1 antibody treatment. WT mice were i.p. injected with αPD-L1 (or IgG), and spleens were collected 3 and 7 days after the treatment and analyzed by multiparametric flow cytometry**.** (**A**) Flow cytometry analyses of Tregs (One-way ANOVA $F_{(2,15)}=7.697$, $**p=0.005$. *Post-hoc* uncorrected Fisher's LSD multiple comparisons between αPD-L1 and IgG groups: $*p<0.05$, $**p<0.01$) and (**B**) their CCR2 expression. (**C**) Flow cytometry analysis of memory CD4$^+$ T cells (One-way ANOVA $F_{(2,15)}=4.374$, $*p=0.0319$. *Post-hoc* uncorrected Fisher's LSD multiple comparisons between αPD-L1 to IgG groups: $*p<0.05$). n=6 mice per group. Data are presented as mean ± s.e.m.
