# Supplementary material for: Key role of the CCR2-CCL2 axis in disease modification in a mouse model of tauopathy
Source: Mol Neurodegener. 2021 Jun 25;16:39. doi: 10.1186/s13024-021-00458-z (PMC8234631; doi:10.1186/s13024-021-00458-z)
Supplement: Supplementary file 5 — Additional file 5 Supplementary Fig. 4. Anti-CCR2 antibody induces reduction in brain infiltrating monocytes following anti-PD-L1 antibody treatment. This experiment included three groups of DM-hTAU mice treated either with: IgG, αPD-L1 or αCCR2 + αPD-L1. αCCR2 was i.p. injected to DM-hTAU mice 3 days prior (Day − 3) to αPD-L1 (Day 0), and then again on days 1, 5 and 9. The brains were analyzed by multiparametric flow cytometry 3 days after the last αCCR2 injection (Day 12). (A) Flow cytometry gating strategy, and (B) quantification of infiltrating monocytes in the brain of DM-hTAU mice. One-way ANOVA F(2,15) = 3.591, p = 0.0532. Post-hoc uncorrected Fisher’s LSD multiple comparisons between the groups: *p < 0.05. n = 6 mice per group. Data are presented as mean ± s.e.m. [file 13024_2021_458_MOESM5_ESM.pdf]

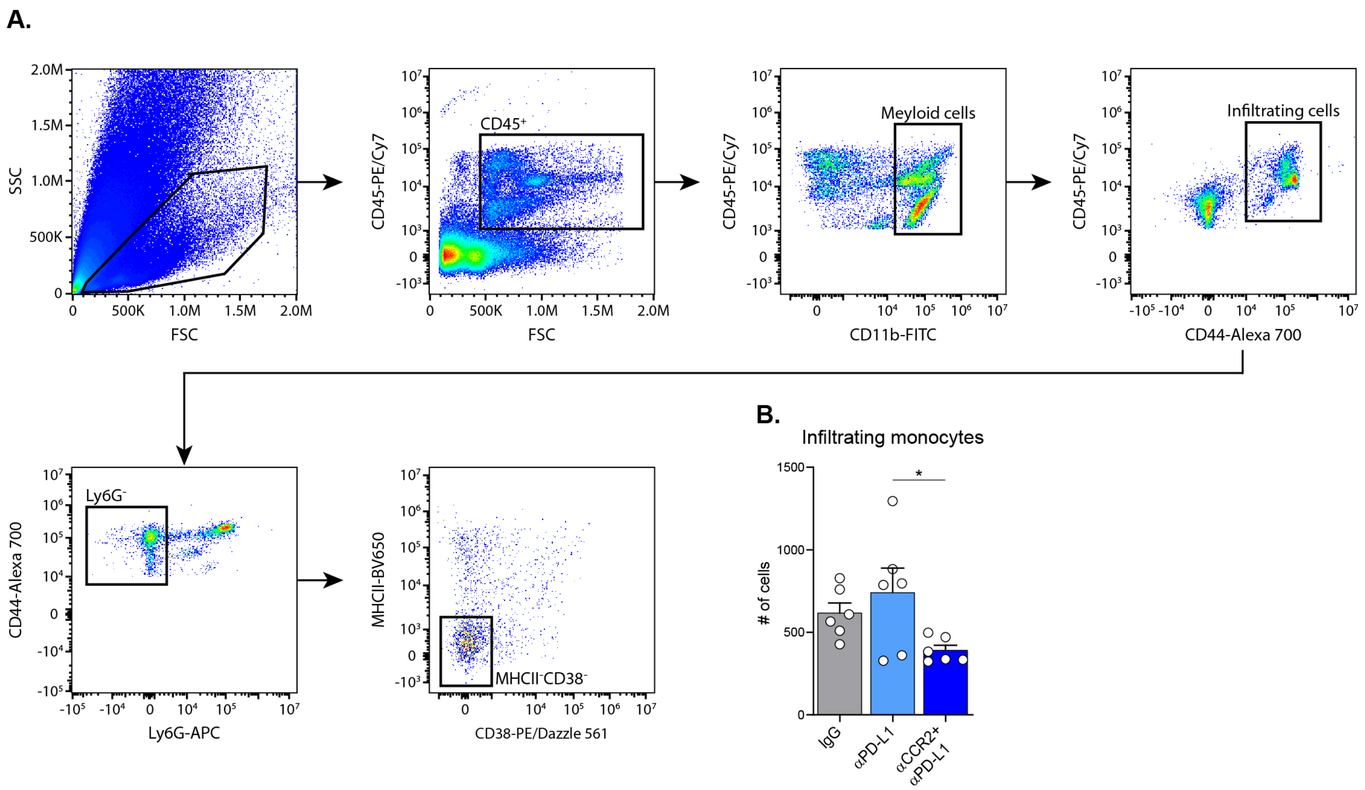

Additional file 5 Supplementary Figure 4. Anti-CCR2 antibody induces reduction in brain infiltrating monocytes following anti-PD-L1 antibody treatment. This experiment included three groups of DM-hTAU mice treated either with: IgG, αPD-L1 or αCCR2+αPD-L1. αCCR2 was i.p. injected to DM-hTAU mice 3 days prior (Day -3) to αPD-L1 (Day 0), and then again on days 1, 5 and 9. The brains were analyzed by multiparametric flow cytometry 3 days after the last αCCR2 injection (Day 12). (**A**) Flow cytometry gating strategy, and (**B**) quantification of infiltrating monocytes in the brain of DM-hTAU mice. One-way ANOVA $F_{(2,15)}=3.591$, $p=0.0532$. *Post-hoc* uncorrected Fisher's LSD multiple comparisons between the groups: *$p<0.05$. n=6 mice per group. Data are presented as mean ± s.e.m.
