# Supplementary material for: Key role of the CCR2-CCL2 axis in disease modification in a mouse model of tauopathy
Source: Mol Neurodegener. 2021 Jun 25;16:39. doi: 10.1186/s13024-021-00458-z (PMC8234631; doi:10.1186/s13024-021-00458-z)
Supplement: Supplementary file 6 — Additional file 6 Supplementary Table 2. The ratio between the number of cells found in the brain following co-administration of αCCR2 and αPD-L1 relative to administration of αPD-L1 alone, was similar for both monocytes and Tregs. [file 13024_2021_458_MOESM6_ESM.pdf]

Additional file 6 Supplementary Table 2

The ratio between the number of cells found in the brain following co-administration of αCCR2 and αPD-L1 relative to administration of αPD-L1 alone, was similar for both monocytes and Tregs.

Ratio	FOXP3$^+$ CD4$^+$ TCRβ$^+$ cells	Ly6C^{hi} monocytes
$\frac{\alpha CCR2+\alpha PD\text{-}L1}{\alpha PD\text{-}L1}$	0.6705	0.6317
